# 8-[(3-Phenyl-1,2,4-oxa­diazol-5-yl)meth­oxy]quinoline monohydrate

**DOI:** 10.1107/S1600536813014529

**Published:** 2013-06-08

**Authors:** Shu-Yuan Bai, Hong Shen, Xin-Yi Han, Ling-Jie Lv, Hai-Bo Wang

**Affiliations:** a College of Food Science and Light Industry, Nanjing University of Technology, Xinmofan Road No. 5 Nanjing, Nanjing 210009, People’s Republic of China; b College of Science, Nanjing University of Technology, Xinmofan Road No. 5 Nanjing, Nanjing 210009, People’s Republic of China

## Abstract

In the title compound, C_18_H_13_N_3_O_2_·H_2_O, the oxa­diazole ring forms dihedral angles 7.21 (10) and 21.25 (11)° with the quinoline and benzene rings, respectively. The crystal structure features O—H⋯N hydrogen bonds and is further consolidated by C—H⋯O hydrogen-bonding inter­actions involving the water molecule of hydration.

## Related literature

For general background, see: Katritzky *et al.* (1992 ▶). For preparation of the title compound, see: Shishue & Henry (1989 ▶). For crystal structure of a related compound, see: Liu *et al.* (2006 ▶).
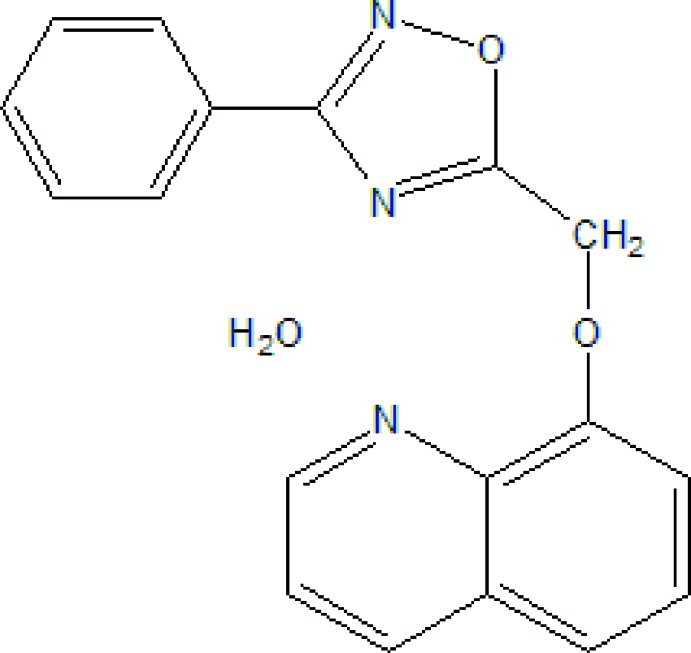


## Experimental

### 

#### Crystal data

C_18_H_13_N_3_O_2_·H_2_O
*M* *_r_* = 321.33
Monoclinic, 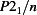

*a* = 7.0430 (14) Å
*b* = 7.5800 (15) Å
*c* = 29.114 (6) Å
β = 95.33 (3)°
*V* = 1547.6 (5) Å^3^
*Z* = 4
Mo *K*α radiation
μ = 0.10 mm^−1^
*T* = 293 K
0.30 × 0.20 × 0.10 mm

#### Data collection

Enraf–Nonius CAD-4 diffractometer
Absorption correction: ψ scan (North *et al.*, 1968 ▶) *T*_min_ = 0.972, *T*_max_ = 0.990
3101 measured reflections
2853 independent reflections
1608 reflections with *I* > 2σ(*I*)
*R*_int_ = 0.025
3 standard reflections every 200 reflections intensity decay: 1%

#### Refinement

*R*[*F* ^2^ > 2σ(*F* ^2^)] = 0.052
*wR*(*F* ^2^) = 0.144
*S* = 0.95
2853 reflections
223 parameters
H atoms treated by a mixture of independent and constrained refinement
Δρ_max_ = 0.15 e Å^−3^
Δρ_min_ = −0.15 e Å^−3^

### 

Data collection: *CAD-4 EXPRESS* (Enraf–Nonius, 1994 ▶); cell refinement: *CAD-4 EXPRESS*; data reduction: *XCAD4* (Harms & Wocadlo, 1995 ▶); program(s) used to solve structure: *SHELXS97* (Sheldrick, 2008 ▶); program(s) used to refine structure: *SHELXL97* (Sheldrick, 2008 ▶); molecular graphics: *SHELXTL* (Sheldrick, 2008 ▶); software used to prepare material for publication: *PLATON* (Spek, 2009 ▶).

## Supplementary Material

Crystal structure: contains datablock(s) global, I. DOI: 10.1107/S1600536813014529/pv2633sup1.cif

Structure factors: contains datablock(s) I. DOI: 10.1107/S1600536813014529/pv2633Isup2.hkl

Supplementary material file. DOI: 10.1107/S1600536813014529/pv2633Isup3.cml

Additional supplementary materials:  crystallographic information; 3D view; checkCIF report

## Figures and Tables

**Table 1:** Hydrogen-bond geometry (Å, °)

*D*—H⋯*A*	*D*—H	H⋯*A*	*D*⋯*A*	*D*—H⋯*A*
O1*W*—H1*W*⋯N2	0.79 (4)	2.20 (4)	2.988 (3)	171 (4)
O1*W*—H2*W*⋯N3	0.81 (4)	2.00 (4)	2.810 (3)	174 (4)
C9—H9*A*⋯O1*W* ^i^	0.97	2.54	3.437 (3)	154
C12—H12*A*⋯O1*W* ^ii^	0.93	2.51	3.268 (4)	139

Symmetry codes: (i) 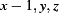; (ii) 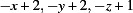.

**Fig. 1.**
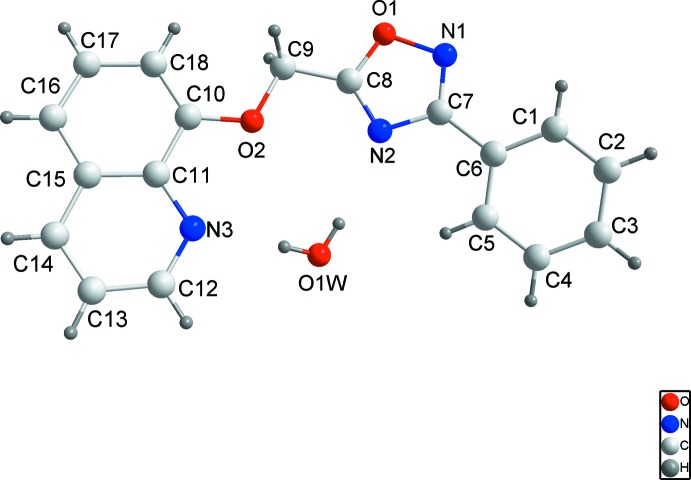
The molecular structure of the title molecule, with the atom-numbering scheme. Displacement ellipsoids are drawn at the 50% probability level.

**Fig. 2.**
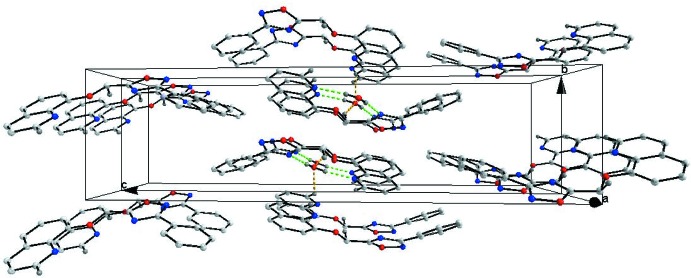
A packing diagram of the title compound viewed down the c axis. Dashed lines indicate intermolecular O—H···N and C—H···O interactions.

## supplementary crystallographic information

### Comment

Due to unique biological activity in medicine and pesticide, 1,2,4-oxadiazole derivatives have received an increased attention. It plays an increasingly important role in pharmaceutical synthesis, if different heterocyclics were introduced into 1,2,4-oxadiazole ring (Katritzky *et al.*, 1992). We have synthesized the title compound which is a novel derivative of 1,2,4-oxadiazole. In this article, we describe the synthesis and crystal structure of the title compound.

In the title molecule (Fig. 1) the bond distances and bond angles agree very well with the corresponding bond distances and angles reported in a closely related compound (Liu *et al.*, 2006). The quinoline ring is essentially planar (rmsd = 0.0118 Å). The oxadiazole ring forms dihedral angles 7.21 (10) and 21.25 (11)° with the quinoline and benzene rings, respectively. The crystal structure is stabilized by O—H···N hydrogen bonds and further consolidated by C—H···O hydrogen bonding interactions involving the water of hydration (Fig. 2 & Tab. 1).

### Experimental

To a flask charged with 30 mL of acetone were added 3-phenyl-5-chloromethyl-1,2,4-oxadiazole (1.95 g, 10 mmol), 8-hydroxy quinoline (1.45 g, 10 mmol), potassium carbonate (2.0 g, 15 mmol), and a catalytic amount of potassium iodide. The reaction mixture was stirred at refluxing condition for about 5 h. After being cooled to room temperature, the mixture was filtered and evaporated in vacuo. The crude product was further recrystallized from ethyl acetate to give white solid (yield = 2.26 g; 75%). Crystals suitable for X-ray crystallographic studies were grown by slow evaporation of ethyl acetate solution.

### Refinement

H atoms were positioned geometrically, with C—H = 0.93, 0.97 and 0.96 Å for aromatic, methylene and methyl H, respectively, and constrained to ride on their parent atoms, with U_iso_(H) = xU_eq_(C,N), where x = 1.5 for methyl H and x = 1.2 for all other H atoms. The H-atoms of the water of hydration were located from a difference map and were allowed to refine with U_iso_(H) = 1.5U_eq_(O).

### Crystal data

**Table:**

C_18_H_13_N_3_O_2_·H_2_O	*F*(000) = 672
*M**_r_* = 321.33	*D*_x_ = 1.379 Mg m^−^^3^
Monoclinic, *P*2_1_/*n*	Melting point: 438 K
Hall symbol: -P 2yn	Mo *K*α radiation, λ = 0.71073 Å
*a* = 7.0430 (14) Å	Cell parameters from 25 reflections
*b* = 7.5800 (15) Å	θ = 9–13°
*c* = 29.114 (6) Å	µ = 0.10 mm^−^^1^
β = 95.33 (3)°	*T* = 293 K
*V* = 1547.6 (5) Å^3^	Block, colourless
*Z* = 4	0.30 × 0.20 × 0.10 mm

### Data collection

**Table:**

Enraf–Nonius CAD-4 diffractometer	1608 reflections with *I* > 2σ(*I*)
Radiation source: fine-focus sealed tube	*R*_int_ = 0.025
Graphite monochromator	θ_max_ = 25.4°, θ_min_ = 1.4°
ω/2θ scans	*h* = 0→8
Absorption correction: ψ scan (North *et al.*, 1968)	*k* = 0→9
*T*_min_ = 0.972, *T*_max_ = 0.990	*l* = −35→34
3101 measured reflections	3 standard reflections every 200 reflections
2853 independent reflections	intensity decay: 1%

### Refinement

**Table:**

Refinement on *F*^2^	Primary atom site location: structure-invariant direct methods
Least-squares matrix: full	Secondary atom site location: difference Fourier map
*R*[*F*^2^ > 2σ(*F*^2^)] = 0.052	Hydrogen site location: inferred from neighbouring sites
*wR*(*F*^2^) = 0.144	H atoms treated by a mixture of independent and constrained refinement
*S* = 0.95	*w* = 1/[σ^2^(*F*_o_^2^) + (0.070*P*)^2^] where *P* = (*F*_o_^2^ + 2*F*_c_^2^)/3
2853 reflections	(Δ/σ)_max_ < 0.001
223 parameters	Δρ_max_ = 0.15 e Å^−^^3^
0 restraints	Δρ_min_ = −0.15 e Å^−^^3^

### Special details

**Table:**

Geometry. All esds (except the esd in the dihedral angle between two l.s. planes) are estimated using the full covariance matrix. The cell esds are taken into account individually in the estimation of esds in distances, angles and torsion angles; correlations between esds in cell parameters are only used when they are defined by crystal symmetry. An approximate (isotropic) treatment of cell esds is used for estimating esds involving l.s. planes.
Refinement. Refinement of F^2^ against ALL reflections. The weighted R-factor wR and goodness of fit S are based on F^2^, conventional R-factors R are based on F, with F set to zero for negative F^2^. The threshold expression of F^2^ > 2sigma(F^2^) is used only for calculating R-factors(gt) etc. and is not relevant to the choice of reflections for refinement. R-factors based on F^2^ are statistically about twice as large as those based on F, and R- factors based on ALL data will be even larger.

### Fractional atomic coordinates and isotropic or equivalent isotropic displacement parameters (Å^2^)

**Table:**

	*x*	*y*	*z*	*U*_iso_*/*U*_eq_	
O1	0.1455 (2)	0.5455 (3)	0.39671 (7)	0.0669 (6)	
O2	0.4113 (2)	0.7022 (2)	0.49830 (6)	0.0521 (5)	
N1	0.2012 (3)	0.5612 (3)	0.35162 (8)	0.0684 (7)	
N2	0.4312 (3)	0.6622 (3)	0.40388 (7)	0.0484 (6)	
N3	0.7080 (3)	0.8663 (3)	0.54211 (7)	0.0501 (6)	
C1	0.3907 (5)	0.7139 (5)	0.27655 (11)	0.0791 (10)	
H1B	0.2625	0.6855	0.2702	0.095*	
C2	0.4931 (7)	0.7816 (5)	0.24221 (11)	0.0948 (12)	
H2B	0.4325	0.8006	0.2129	0.114*	
C3	0.6821 (6)	0.8209 (5)	0.25099 (12)	0.0874 (11)	
H3B	0.7491	0.8687	0.2279	0.105*	
C4	0.7724 (5)	0.7900 (5)	0.29354 (11)	0.0843 (11)	
H4A	0.9022	0.8128	0.2991	0.101*	
C5	0.6733 (4)	0.7253 (4)	0.32851 (10)	0.0694 (9)	
H5A	0.7360	0.7061	0.3576	0.083*	
C6	0.4811 (4)	0.6888 (3)	0.32043 (9)	0.0550 (7)	
C7	0.3704 (4)	0.6317 (4)	0.35832 (9)	0.0518 (7)	
C8	0.2890 (3)	0.6098 (3)	0.42497 (9)	0.0478 (7)	
C9	0.2552 (3)	0.6131 (4)	0.47439 (9)	0.0507 (7)	
H9A	0.1371	0.6743	0.4785	0.061*	
H9B	0.2462	0.4937	0.4860	0.061*	
C10	0.4014 (3)	0.7312 (3)	0.54418 (9)	0.0446 (6)	
C11	0.5607 (3)	0.8199 (3)	0.56692 (9)	0.0436 (6)	
C12	0.8557 (4)	0.9462 (4)	0.56418 (10)	0.0587 (8)	
H12A	0.9559	0.9794	0.5474	0.070*	
C13	0.8705 (4)	0.9839 (4)	0.61116 (11)	0.0652 (8)	
H13A	0.9782	1.0402	0.6251	0.078*	
C14	0.7261 (4)	0.9374 (4)	0.63636 (10)	0.0639 (8)	
H14A	0.7340	0.9610	0.6678	0.077*	
C15	0.5641 (4)	0.8531 (3)	0.61456 (9)	0.0496 (7)	
C16	0.4096 (4)	0.7993 (4)	0.63873 (10)	0.0627 (8)	
H16A	0.4103	0.8221	0.6701	0.075*	
C17	0.2598 (4)	0.7139 (4)	0.61586 (10)	0.0627 (8)	
H17A	0.1585	0.6781	0.6320	0.075*	
C18	0.2546 (4)	0.6787 (3)	0.56860 (10)	0.0527 (7)	
H18A	0.1508	0.6194	0.5537	0.063*	
O1W	0.8011 (3)	0.7785 (4)	0.45314 (8)	0.0770 (7)	
H1W	0.709 (6)	0.747 (5)	0.4376 (13)	0.116*	
H2W	0.770 (5)	0.810 (5)	0.4781 (14)	0.116*	

### Atomic displacement parameters (Å^2^)

**Table:**

	*U*^11^	*U*^22^	*U*^33^	*U*^12^	*U*^13^	*U*^23^
O1	0.0498 (11)	0.0858 (15)	0.0641 (13)	−0.0154 (11)	−0.0007 (10)	−0.0105 (11)
O2	0.0398 (9)	0.0638 (12)	0.0531 (11)	−0.0111 (9)	0.0062 (8)	−0.0030 (9)
N1	0.0558 (15)	0.0858 (19)	0.0621 (17)	−0.0069 (14)	−0.0027 (12)	−0.0117 (14)
N2	0.0456 (12)	0.0520 (14)	0.0465 (13)	−0.0002 (11)	−0.0010 (10)	0.0055 (10)
N3	0.0407 (12)	0.0477 (13)	0.0628 (15)	−0.0052 (11)	0.0094 (11)	−0.0013 (11)
C1	0.084 (2)	0.095 (3)	0.055 (2)	0.007 (2)	−0.0086 (18)	−0.0022 (18)
C2	0.136 (4)	0.100 (3)	0.047 (2)	0.013 (3)	0.000 (2)	0.0069 (19)
C3	0.125 (3)	0.083 (3)	0.058 (2)	−0.009 (2)	0.025 (2)	−0.0040 (19)
C4	0.093 (2)	0.103 (3)	0.060 (2)	−0.021 (2)	0.0226 (19)	−0.008 (2)
C5	0.072 (2)	0.086 (2)	0.0511 (18)	−0.0084 (18)	0.0096 (15)	−0.0025 (16)
C6	0.0674 (18)	0.0506 (17)	0.0458 (16)	0.0046 (15)	−0.0015 (14)	−0.0047 (13)
C7	0.0496 (16)	0.0559 (17)	0.0480 (17)	0.0047 (14)	−0.0057 (13)	−0.0052 (13)
C8	0.0388 (14)	0.0489 (16)	0.0543 (17)	0.0007 (13)	−0.0035 (13)	0.0024 (13)
C9	0.0319 (13)	0.0548 (17)	0.0644 (18)	−0.0057 (13)	−0.0010 (12)	0.0031 (14)
C10	0.0417 (14)	0.0441 (15)	0.0484 (15)	0.0021 (12)	0.0070 (12)	0.0027 (12)
C11	0.0392 (14)	0.0393 (14)	0.0530 (16)	0.0044 (12)	0.0075 (12)	0.0026 (12)
C12	0.0444 (16)	0.0544 (18)	0.077 (2)	−0.0062 (14)	0.0029 (14)	−0.0024 (16)
C13	0.0529 (17)	0.064 (2)	0.075 (2)	0.0011 (16)	−0.0135 (16)	−0.0144 (17)
C14	0.0683 (19)	0.063 (2)	0.0590 (18)	0.0127 (17)	−0.0046 (16)	−0.0103 (16)
C15	0.0479 (15)	0.0474 (16)	0.0530 (17)	0.0067 (13)	0.0016 (13)	−0.0006 (13)
C16	0.071 (2)	0.070 (2)	0.0493 (16)	0.0152 (17)	0.0170 (15)	0.0014 (15)
C17	0.0569 (18)	0.071 (2)	0.063 (2)	0.0039 (16)	0.0227 (15)	0.0065 (16)
C18	0.0430 (14)	0.0549 (17)	0.0615 (18)	−0.0011 (13)	0.0124 (13)	0.0059 (14)
O1W	0.0487 (12)	0.1114 (19)	0.0729 (16)	−0.0250 (13)	0.0159 (11)	−0.0162 (14)

### Geometric parameters (Å, º)

**Table:**

O1—C8	1.335 (3)	C8—C9	1.480 (3)
O1—N1	1.410 (3)	C9—H9A	0.9700
O2—C10	1.362 (3)	C9—H9B	0.9700
O2—C9	1.417 (3)	C10—C18	1.367 (3)
N1—C7	1.304 (3)	C10—C11	1.418 (3)
N2—C8	1.285 (3)	C11—C15	1.408 (3)
N2—C7	1.375 (3)	C12—C13	1.392 (4)
N3—C12	1.318 (3)	C12—H12A	0.9300
N3—C11	1.364 (3)	C13—C14	1.355 (4)
C1—C2	1.385 (5)	C13—H13A	0.9300
C1—C6	1.387 (4)	C14—C15	1.406 (4)
C1—H1B	0.9300	C14—H14A	0.9300
C2—C3	1.365 (5)	C15—C16	1.410 (4)
C2—H2B	0.9300	C16—C17	1.358 (4)
C3—C4	1.359 (5)	C16—H16A	0.9300
C3—H3B	0.9300	C17—C18	1.399 (4)
C4—C5	1.377 (4)	C17—H17A	0.9300
C4—H4A	0.9300	C18—H18A	0.9300
C5—C6	1.380 (4)	O1W—H1W	0.79 (4)
C5—H5A	0.9300	O1W—H2W	0.81 (4)
C6—C7	1.473 (4)		
			
C8—O1—N1	106.44 (19)	O2—C9—H9B	110.2
C10—O2—C9	116.82 (19)	C8—C9—H9B	110.2
C7—N1—O1	103.0 (2)	H9A—C9—H9B	108.5
C8—N2—C7	102.9 (2)	O2—C10—C18	125.0 (2)
C12—N3—C11	117.7 (2)	O2—C10—C11	115.1 (2)
C2—C1—C6	119.4 (3)	C18—C10—C11	119.9 (3)
C2—C1—H1B	120.3	N3—C11—C15	122.1 (2)
C6—C1—H1B	120.3	N3—C11—C10	118.9 (2)
C3—C2—C1	120.7 (3)	C15—C11—C10	119.0 (2)
C3—C2—H2B	119.7	N3—C12—C13	123.8 (3)
C1—C2—H2B	119.7	N3—C12—H12A	118.1
C4—C3—C2	119.9 (4)	C13—C12—H12A	118.1
C4—C3—H3B	120.0	C14—C13—C12	119.2 (3)
C2—C3—H3B	120.0	C14—C13—H13A	120.4
C3—C4—C5	120.6 (4)	C12—C13—H13A	120.4
C3—C4—H4A	119.7	C13—C14—C15	119.5 (3)
C5—C4—H4A	119.7	C13—C14—H14A	120.3
C4—C5—C6	120.1 (3)	C15—C14—H14A	120.3
C4—C5—H5A	119.9	C14—C15—C11	117.7 (3)
C6—C5—H5A	119.9	C14—C15—C16	122.5 (3)
C5—C6—C1	119.2 (3)	C11—C15—C16	119.8 (3)
C5—C6—C7	120.7 (2)	C17—C16—C15	119.6 (3)
C1—C6—C7	120.0 (3)	C17—C16—H16A	120.2
N1—C7—N2	114.3 (3)	C15—C16—H16A	120.2
N1—C7—C6	123.2 (2)	C16—C17—C18	121.4 (3)
N2—C7—C6	122.3 (2)	C16—C17—H17A	119.3
N2—C8—O1	113.4 (2)	C18—C17—H17A	119.3
N2—C8—C9	131.5 (2)	C10—C18—C17	120.3 (3)
O1—C8—C9	115.1 (2)	C10—C18—H18A	119.9
O2—C9—C8	107.3 (2)	C17—C18—H18A	119.9
O2—C9—H9A	110.2	H1W—O1W—H2W	109 (4)
C8—C9—H9A	110.2		
			
C8—O1—N1—C7	0.3 (3)	C9—O2—C10—C18	1.0 (4)
C6—C1—C2—C3	1.1 (6)	C9—O2—C10—C11	179.7 (2)
C1—C2—C3—C4	1.4 (6)	C12—N3—C11—C15	−0.6 (4)
C2—C3—C4—C5	−2.4 (6)	C12—N3—C11—C10	−179.0 (2)
C3—C4—C5—C6	0.9 (5)	O2—C10—C11—N3	−0.7 (3)
C4—C5—C6—C1	1.6 (5)	C18—C10—C11—N3	178.1 (2)
C4—C5—C6—C7	−175.6 (3)	O2—C10—C11—C15	−179.1 (2)
C2—C1—C6—C5	−2.6 (5)	C18—C10—C11—C15	−0.3 (4)
C2—C1—C6—C7	174.6 (3)	C11—N3—C12—C13	0.7 (4)
O1—N1—C7—N2	0.6 (3)	N3—C12—C13—C14	−0.2 (4)
O1—N1—C7—C6	−174.3 (2)	C12—C13—C14—C15	−0.3 (4)
C8—N2—C7—N1	−1.2 (3)	C13—C14—C15—C11	0.4 (4)
C8—N2—C7—C6	173.7 (2)	C13—C14—C15—C16	179.2 (3)
C5—C6—C7—N1	−165.4 (3)	N3—C11—C15—C14	0.1 (4)
C1—C6—C7—N1	17.5 (4)	C10—C11—C15—C14	178.4 (2)
C5—C6—C7—N2	20.2 (4)	N3—C11—C15—C16	−178.8 (2)
C1—C6—C7—N2	−157.0 (3)	C10—C11—C15—C16	−0.4 (4)
C7—N2—C8—O1	1.4 (3)	C14—C15—C16—C17	−178.1 (3)
C7—N2—C8—C9	−176.7 (3)	C11—C15—C16—C17	0.8 (4)
N1—O1—C8—N2	−1.1 (3)	C15—C16—C17—C18	−0.3 (4)
N1—O1—C8—C9	177.3 (2)	O2—C10—C18—C17	179.4 (2)
C10—O2—C9—C8	175.3 (2)	C11—C10—C18—C17	0.7 (4)
N2—C8—C9—O2	4.4 (4)	C16—C17—C18—C10	−0.4 (4)
O1—C8—C9—O2	−173.7 (2)		

### Hydrogen-bond geometry (Å, º)

**Table:**

*D*—H···*A*	*D*—H	H···*A*	*D*···*A*	*D*—H···*A*
O1*W*—H1*W*···N2	0.79 (4)	2.20 (4)	2.988 (3)	171 (4)
O1*W*—H2*W*···N3	0.81 (4)	2.00 (4)	2.810 (3)	174 (4)
C9—H9*A*···O1*W*^i^	0.97	2.54	3.437 (3)	154
C12—H12*A*···O1*W*^ii^	0.93	2.51	3.268 (4)	139

Symmetry codes: (i) *x*−1, *y*, *z*; (ii) −*x*+2, −*y*+2, −*z*+1.

## References

[bb1] Enraf–Nonius (1994). *CAD-4 EXPRESS* Enraf–Nonius, Delft, The Netherlands.

[bb2] Harms, K. & Wocadlo, S. (1995). *XCAD4* University of Marburg, Germany.

[bb3] Katritzky, A. R., Ji, F. B. & Fan, W. Q. (1992). *Heterocycl. Chem.* **29**, 1519–1523.

[bb4] Liu, Z.-Q., Wang, H.-B., Pu, Y.-Q. & Yan, X.-C. (2006). *Acta Cryst.* E**62**, o1131–o1132.

[bb5] North, A. C. T., Phillips, D. C. & Mathews, F. S. (1968). *Acta Cryst.* A**24**, 351–359.

[bb6] Sheldrick, G. M. (2008). *Acta Cryst.* A**64**, 112–122. 10.1107/S0108767307043930 18156677

[bb7] Shishue, C. & Henry, J. S. (1989). *J Heterocycl. Chem* **26**, 125–128.

[bb8] Spek, A. L. (2009). *Acta Cryst.* D**65**, 148–155. 10.1107/S090744490804362X PMC2631630 19171970

